# Acute Myeloid Leukemia with Normal Cytogenetics and *NPM1*-Mutation: Impact of Mutation Topography on Outcomes

**DOI:** 10.3390/biomedicines12122921

**Published:** 2024-12-23

**Authors:** Mingyue Zhao, Mingyue Liao, Robert Peter Gale, Meijie Zhang, Lixin Wu, Nan Yan, Lixia Liu, Jiayue Qin, Shanbo Cao, Yingjun Chang, Qian Jiang, Lanping Xu, Xiaohui Zhang, Xiaojun Huang, Hao Jiang, Guorui Ruan

**Affiliations:** 1 Beijing Key Laboratory of Hematopoietic Stem Cell Transplantation, National Clinical Research Center for Hematologic Disease, Peking University Institute of Hematology, Peking University People’s Hospital, Beijing 100044, China; zhaomingyue617@163.com (M.Z.); liaomy000@163.com (M.L.); rgale@celgene.com (R.P.G.); wulixinsea@foxmail.com (L.W.); ynxy9512@126.com (N.Y.); rmcyj@bjmu.edu.cn (Y.C.); jiangqian@medmail.com.cn (Q.J.); lpxu_0415@sina.com (L.X.); zhangxh100@sina.com (X.Z.); huangxiaojun@bjmu.edu.cn (X.H.); 2 Centre for Haematology, Department of Immunology and Inflammation, Imperial College of Science, Technology and Medicine, London SW7 2AZ, UK; 3 Division of Biostatistics, Medical College of Wisconsin, IBMTR/ABMTR, Milwaukee, WI 53226, USA; meijie@mcw.edu; 4 Acornmed Biotechnology Co., Ltd., Floor 18, Block 5, Yard 18, Kechuang 13 RD, Beijing 100176, China; lixialiu@acornmed.com (L.L.); jyqin@live.cn (J.Q.); shanbocao@acornmed.com (S.C.); 5 Peking-Tsinghua Center for Life Sciences, Academy for Advanced Interdisciplinary Studies, Peking University, Beijing 100091, China

**Keywords:** acute myeloid leukemia, normal cytogenetics, *NPM1* mutation, risk stratification

## Abstract

**Background**: About half of adults with acute myeloid leukemia with normal cytogenetics (CN-AML) have *NPM1* mutations. There is controversy regarding their prognosis and best therapy. **Methods**: We studied 150 subjects with these features using targeted regional sequencing. Prognostic stratification was carried out based on risk factors, and we assessed the effects of two post-remission strategies with and without transplant across risk cohorts. **Results**: In multi-variable analyses, a positive MRD test after the second consolidation cycle (HR = 6.00; 95% CI [3.31, 10.85]; *p* < 0.001), *DNMT3A* mutations (HR = 3.01 [1.57, 5.78]; *p* < 0.001), *FLT3*-ITD mutation with high variant allele frequency (HR = 4.40 [1.89, 10.24]; *p* < 0.001) and *DDX11* mutations (HR = 4.38 [2.38, 8.04]; *p* < 0.001) were independently correlated with higher cumulative incidence of relapse (CIR) and worse leukemia-free survival (LFS) (HR = 5.49 [3.01, 10.04]; *p <* 0.001; HR = 2.99 [1.60, 5.62]; *p <* 0.001; HR = 4.20 [1.87, 9.40]; *p <* 0.001; and HR = 4.22, 95% CI [1.99, 8.95], *p <* 0.001). Subjects with ≥1 high-risk co-variate who received a transplant had a lower CIR and better LFS, whereas others did not. **Conclusions**: We identified co-variates associated with CIR and LFS in subjects of *NPM1*-mutated CN-AML.

## 1. Introduction

Mutations in nucleophosmin 1 (*NPM1*), FMS-like tyrosine kinase 3 (*FLT3*) and CCAAT/enhancer-binding protein α (*CEBPA*) contribute to risk stratification of cytogenetically normal acute myeloid leukemia (CN-AML) [1,2,3]. *NPM1* mutation is reported in 25–35% of adults with AML and an even higher percentage of those with normal cytogenetics, 45–64% [4]. AML with *NPM1* mutation was first recognized as a distinct entity in the 2008 World Health Organization (WHO) classification system and carried forth in subsequent editions [5].

The distinguishing feature of *NPM1* mutation is aberrant cytoplasmic localization [6]. The *NPM1* mutation appears to be a late event in leukemia development and is usually associated with co-mutations such as DNA methyltransferase 3 alpha (*DNMT3A*), Fms-like tyrosine kinase 3 internal tandem duplication (*FLT3*-ITD) and isocitrate dehydrogenase (NADP(+)1/2) (*IDH1/2*), which also affect prognosis [2,3].

People with *NPM1/*NRAS proto-oncogene GTPase (*NRAS*), *NPM1/RAD21* cohesin complex component (*RAD21*), or *NPM1/FLT3*-tyrosine kinase point mutation (*FLT3*-TKD) genotypes have relatively good outcomes, whereas those with *NPM1/*WT1 transcription factor (*WT1*)-mutated or *NPM1*/*FLT3*-ITD/*DNMT3A*-mutated genotypes have adverse outcomes [3,7,8,9].

These complexities make it important to consider co-mutations in subjects with *NPM1* mutations to predict outcomes. Our aim was to utilize high-depth targeted regional sequencing (TRS) technology to detect more myeloid-cancer-related mutations, combined with clinical data, to estimate the prognosis of adults with CN-AML harboring *NPM1* mutations.

## 2. Materials and Methods

### 2.1. Subjects and Treatment Protocols

**Figure 1:**
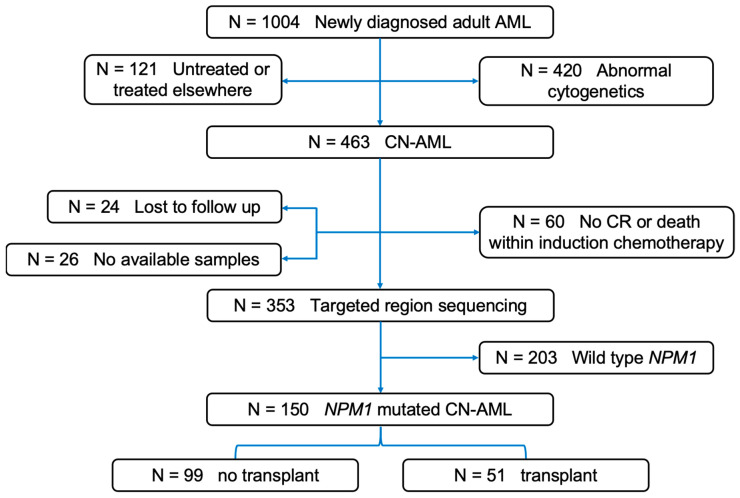
Subject recruitment and cohort assignment. AML, acute myeloid leukemia; CN, cytogenetically normal; CR, complete response.

A total of 1004 consecutive newly diagnosed, untreated persons with AML were studied from January 2012 to December 2019 at Peking University People’s Hospital (China). AML was diagnosed using the WHO 2022 criteria [5]. Cytogenetic studies were carried out using standard techniques. Targeted regional sequencing was carried out on a cohort of 150 consecutive subjects with *NPM1*-mutated CN-AML who presented the following characteristics: (1) age > 15 years; and (2) achieved a histological complete remission with or without normalization of blood cell concentrations (Figure 1).

The protocols for induction and consolidation therapies were reported [10,11,12]. After the second consolidation cycle, the physicians assessed the risks and benefits of the patient receiving an allotransplant versus continuing with consolidation chemotherapy. Allogeneic hematopoietic stem cell transplantation (allo-HSCT) was carried out as reported [10,11,12].

### 2.2. Response Definitions

Histological complete response (CR) was defined as <5 percent bone marrow blasts. Complete hematological recovery was defined as blood neutrophils > 1.0 × 10^9^/L, platelet concentration > 100 × 10^9^/L without platelet transfusions, and no RBC transfusions. Relapse was defined as >5 percent bone marrow blasts, blasts in the blood or extra-medullary leukemia.

### 2.3. Measurable Residual Disease Testing

Measurable residual disease (MRD) testing was performed using multi-parameter flow cytometry (MPFC) with a panel of eight antibody combinations that recognize CD7, CD11b, CD13, CD14, CD16, CD19, CD33, CD34, CD38, CD41, CD45, CD56, CD61, CD64, CD71, CD117, CD123, and HLA-DR. After each chemotherapy cycle, bone marrow samples were tested for MRD based on the leukemia-associated immune phenotype (LAIP) identified at diagnosis. An MRD test result ≥ 0.01 percent was scored as positive.

### 2.4. High-Depth Targeted Regional Sequencing and Analyses

High-depth targeted region sequencing (TRS) analysis was performed on bone marrow samples from subjects, focusing on 236 genes (Supplement Table S1) known or considered to be potential mutation hotspots in hematological cancers. DNA was extracted using DNAzol^®^ kits (Invitrogen, Carlsbad, CA, USA) using the manufacturers’ instructions. Sequencing details were reported [12]. The following criteria were used to filter raw variant results: (1) average effective sequencing depth on target per sample ≥ 1000×; (2) mapping quality ≥ 30; (3) base quality ≥ 30; and (4) variant allele frequency (VAF) ≥ 1% for SNVs and small indels. Burrows–Wheeler alignment (BWA version 0.7.12) was used to align the trimmed reads. The MarkDuplicates tool from Picard was used to mark the PCR duplicates. Indel Realigner and Base Recalibrator from Genome Analysis Toolkit (GATK version 3.8) were applied for re-alignment and re-calibration of the BWA data. Variant calling, including SNVs and small indels, was carried out in Mutect2. ANNOVAR software (V2020) was used to annotate all the variants including 1000 G projects, COSMIC, SIFT and PolyPhen. Typical and atypical *NPM1* mutations (type A/B/D) were detected by real-time quantitative polymerase chain reaction (RT-qPCR) and first-generation sequencing [13]. *FLT3*-ITD mutations were detected by capillary electrophoresis fragment analyses.

### 2.5. Endpoints and Statistical Methods

The last follow-up was 31 December 2020. The primary endpoint was cumulative incidence of relapse (CIR) measured from CR. The secondary endpoint was leukemia-free survival (LFS) defined as the survival period with continuous CR from CR1. Subjects receiving a transplant were censored at the time of transplantation when evaluating co-variates for CIR and LFS.

Categorical variables were expressed in percentages and analyzed by Pearson’s Chi-square test. Normally distributed continuous variables were described as mean (standard deviation) values and analyzed by independent-sample *t*-test. Non-normally distributed continuous variables were described as median (range) values and analyzed by the Mann–Whitney U test. Homogeneity of variances between groups was assessed using the Levene test. CIR was calculated using competing risks with non-relapse mortality (NRM), and the Fine–Gray test was used for univariate analysis. The LFS rate was calculated using the Kaplan–Meier method with the log-rank test. Competing risks model and multi-variate Cox regression analyses were performed for the co-variates selected by the univariate analyses with a criterion of *p* < 0.10. The variance inflation factor (VIF) was estimated to check for multi-collinearity amongst co-variates included in the multi-variate analyses. Kyoto Encyclopedia of Genes and Genomes (KEGG) enrichment analysis for the mutational genes was carried out by using the R ClusterProfiler package. Analyses were performed using SPSS software version 25.0 (Chicago, IL, USA) and R software version 4.1.0. (http://www.Rproject.org). *p* < 0.05 was considered significant.

### 2.6. Trial Registration

This trial is registered at Clinicaltrials.gov (NCT01455272, NCT02185261) and in chictr.org (ChiCTR-OCH-10000940).

## 3. Results

### 3.1. Subject Baseline Co-Variates and CIR or LFS

Subject co-variates are displayed in Table 1. For post-remission therapy, 99 did not received a transplant and 51 received a transplant. The transplant cohort was younger, with medians of 33 years (Interquartile Range, IQR, 18 years) versus 53 years (IQR, 19 years; *p* < 0.001). Other co-variates were similar. Five-year CIR and LFS in the no-transplant cohort were 60% and 36%, compared to 28% and 60% (HR 0.44, 95% CI [0.24, 0.82], *p* = 0.01; HR 0.59, 95% CI [0.35, 1.01], *p* = 0.05) in the transplant cohort. (Supplement Figure S1).

### 3.2. Genomic Analyses and Mutation Topography

**Figure 2:**
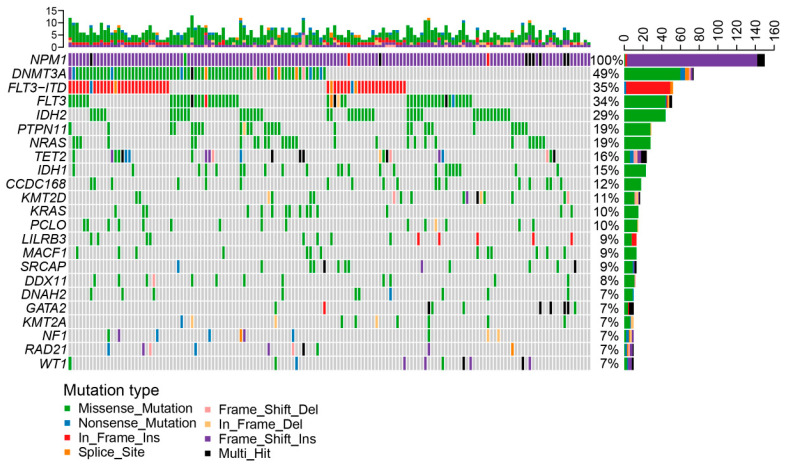
Genomic landscape. Genes mutated in ≥10 subjects are shown. Each column indicates data of one sample; each row represents a gene. Mutated genes were color-coded for mis-sense (green), non-sense (blue), in-frame ins (red), splice-site (orange), frameshift del (pink), in-frame del (yellow), frame-shift ins (purple) and multi-hit (black). The top bar indicates mutation load (mutation/Mb DNA), and the right bar indicates mutation frequency.

One subject had a mis-sense and two in-frame insertion mutations. Eight had >1 type of *NPM1* mutation referred to multi-hit (Figure 2). A total of 771 other mutations (not all 236 genes in the panel were found to have mutations) were detected, with a median of five (Range: 1–12) per subject, and >5 mutations were detected in 56 subjects. Mis-sense mutations were the most common (N = 564) followed by frame-shift insertions (N = 182) and in-frame insertions (N = 64). Mutations in ≥10 subjects were divided into eight subgroups: (1) signaling pathway (*FLT3*, *FLT3*-ITD, *PTPN11*, *NARS*, *KRAS*, *LILRB3, MACF1* and *NF1*); (2) DNA methylation (*DNMT3A*, *TET2*, *IDH1* and *IDH2*); (3) histone methylation (*KMT2A* and *KMT2D*); (4) cohesion complex (*DDX11* and *RAD21*); (5) chromatin modifiers (*SRCAP*); (6) transcription factors (*GATA2*); (7) tumor suppressors (*WT1*); (8) and others (*CCDC168*, *PCLO*, *DNAH2*).

*DNMT3A* (N = 74) was the most frequently mutated gene followed by *FLT3*-ITD (N = 52, 34.67%), *FLT3* (*FLT3* mutation types other than *FLT3*-ITD, N = 51, 34%), and *IDH2* (N = 44, 29.33%). Median variable allele frequency (VAF) was 0.43 (Range, 0.01–1.00). The VAFs of mutations in the DNA methylation pathways and histone methylation were significantly higher compared with those of mutations in the activated signaling pathways (0.43 [0.01, 0.92] versus 0.17 [0.01, 0.78]; *p* < 0.001; 0.44 [0.01, 0.60] versus 0.17 [0.01, 0.78], *p* < 0.01; Supplement Figure S2).

We identified 10 pairs of co-occurring genes and 5 pairs of mutually exclusive mutations, which were statistically significant. *RAD21*, *NRAS*, and *PTPN11* were co-mutations, whereas *IDH2* and *TET2* mutations, and *PTPN11* and *FLT3*-ITD mutations were mutually exclusive mutations (Supplement Figure S3A). Using KEGG pathway enrichment analysis, we found that co-mutations in CN-AML with *NPM1* mutations were related to cancer and metabolism (Supplement Table S2, Supplement Figure S3B). Associations between mutations and subject-related co-variates at diagnosis are shown in Table 2. *DNMT3A* mutations were associated with increased age and high WBC and platelet concentrations at diagnosis. *FLT3*-ITD mutations were associated with a higher percentage of bone marrow blasts.

### 3.3. Prognostic Co-Variates

Clinical variables and gene mutations occurring in ≥10 patients were included in the univariate analyses and six variables with *p* < 0.10 were eligible for subsequent analyses (Supplement Table S3). Considering the interaction between MRD test results after the first and second consolidation cycles and referring to previous reports [14], we used a backward elimination process and ultimately included the MRD test results after the second consolidation cycle as a co-variate.

Eventually, four co-variates were significantly correlated with CIR in multi-variable analyses: (1) positive MRD test after the second consolidation cycle (HR = 6.00 [3.31, 10.85]; *p* < 0.001), (2) *DNMT3A* mutation (HR = 3.01 [1.57, 5.78], *p* < 0.001); (3) *FLT3*-ITD mutation with high VAF (≥0.5) (HR = 4.40 [1.89, 10.24]; *p* < 0.001) and (4) DEAD/H-box helicase 11 (*DDX11*) mutation (HR = 4.38 [2.38, 8.04]; *p <* 0.001) and LFS (HR = 5.49 [3.01, 10.04]; *p <* 0.001; HR = 2.99 [1.60, 5.62]; *p <* 0.001; HR = 4.20 [1.87, 9.40]; *p <* 0.001; and HR = 4.22, 95% CI [1.99, 8.95], *p <* 0.001). (Table 3). The VIFs of the co-variates included in the multi-variate analyses were below five, suggesting no significant multi-collinearity among the co-variates.

For the newly discovered *DDX11* mutations, we found that, compared to wild-type subjects, those with the *DDX11* mutations had a significantly higher CIR and a notably shorter LFS (Supplement Figure S4).

### 3.4. Impact of Transplants

**Figure 3:**
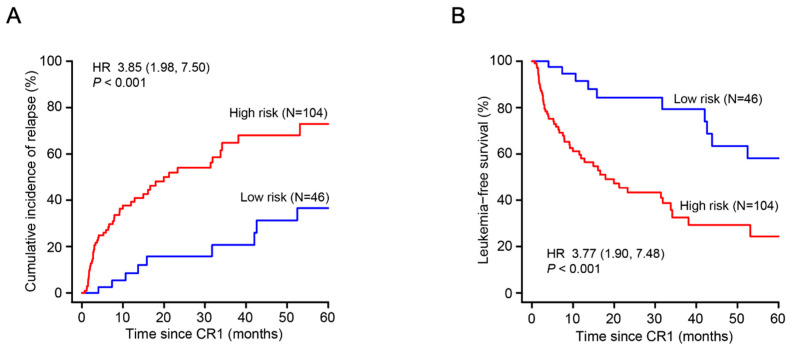
Cumulative incidence of relapse (**A**) and leukemia-free survival (**B**) in the high-risk and low-risk cohort.

We assigned subjects with ≥1 adverse risk co-variates to a high-risk cohort (N = 104) and others to a low-risk cohort (N = 46) with corresponding 5-year CIRs of 73% [58, 88%] and 37% [16, 57%]; (HR = 3.85 [1.98, 7.50]; *p* < 0.001) and 5-year LFSs of 24% [14, 43%] and 58% [41, 84%] (HR = 3.77 [1.90, 7.48]; *p* < 0.001) (Figure 3).

**Figure 4:**
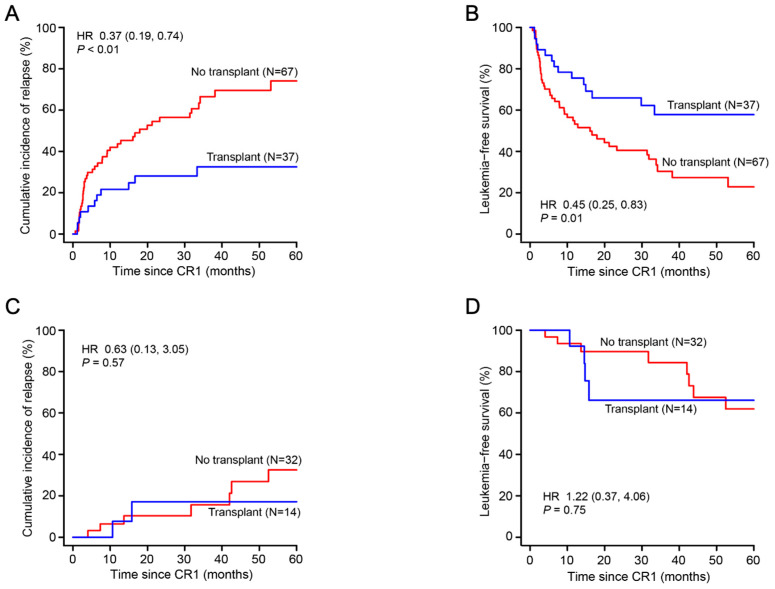
Cumulative incidence of relapse and leukemia-free survival in the transplant and no-transplant subjects of high-risk cohort (**A**,**B**) and low-risk cohort (**C**,**D**).

Transplant recipients in the high-risk cohort (N = 37) had a lower 5-year CIR, 33% [16, 49%] versus 74% [60, 89%] (HR = 0.37 [0.19, 0.74]; *p* < 0.01), and a better 5-year LFS, 58% [43, 78%] versus 23% [13, 41%] (HR = 0.45 [0.25, 0.83]; *p* = 0.01), compared with those in the no-transplant cohort (N = 67). In the low-risk cohort, 14 subjects received a transplant and 32 did not receive a transplant. Five-year CIRs were 17% [0, 40%] versus 32% [11, 54%] (HR = 0.63 [0.13, 3.05]; *p* = 0.57), and five-year LFSs were 66% [44, 100%] versus 62% [44, 88%] (HR = 1.22 [0.37, 4.06]; *p* = 0.75) (Figure 4).

## 4. Discussion

We found that in subjects with CN-AML and *NPM1* mutation, those with ≥1 high-risk co-variate, including positive MRD test after the second consolidation cycle, *DNMT3A* mutation, *FLT3*-ITD mutation with high VAF and *DDX11* mutation, had a higher CIR and worse LFS compared to subjects without these co-variates. We also found a lower CIR and better LFS in subjects in the high-risk cohort who received a transplant, which was not observed in the low-risk cohort.

Compared to the recent study published by Yao et al. [15], our research revealed that the mutation landscape of *NPM1* mutations in CN-AML subjects was similar to their findings. We also identified *DNMT3A* and *FLT3*-ITD mutations as poor prognostic factors. However, their study did not emphasize that the subjects were cytogenetically normal AML patients. Furthermore, we discovered a novel *DDX11* mutation that adversely affects prognosis. In addition, we considered the impact of MRD as a clinical factor on prognosis.

*DNMT3A*, a protein responsible for DNA methylation, is mutated in approximately 20% of all AML cases. It commonly co-mutates with *NPM1* in 60% of cases, and in about 30% of cases, both *NPM1* and *FLT3* mutations are present [16,17,18,19,20]. Compared to wild-type *DNMT3A* subjects, those with *DNMT3A* mutations tend to be older, have higher WBC concentration, and more frequently have normal karyotype at diagnosis [16,18,19,21,22]. Although several studies reported that *DNMT3A* mutations were independently associated with poor outcomes in AML, especially in CN-AML [18,21,23,24], the impact of *DNMT3A* mutations in clinical decision-making remains debatable [19,20,25]. In our study, *DNMT3A* mutations were the most frequently co-occurring event in *NPM1*-mutated CN-AML subjects (49.33%). These subjects were older and had a higher WBC concentration, which was consistent with previous studies [16,17,18,19,21,22]. We further identified *DNMT3A* mutations as one of the poor prognostic factors, and subjects harboring *DNMT3A* mutations were classified into high-risk cohort, for whom allo-HSCT could improve survival.

*FLT3*-ITD has consistently been associated with high WBC and BM blast concentration, an increased risk for relapse, and inferior survival [26,27,28]. In the 2022 ELN guidelines [29], the *FLT3*-ITD allelic ratio is no longer considered in risk classification. This change reflects the impact of FLT3 inhibitor use [30] on the natural course of AML with *FLT3*-ITD mutations and the increasing importance of MRD in guiding treatment decisions [29]. In our study, subjects with *FLT3*-ITD mutations with high VAF were classified into the high-risk cohort and may benefit from allo-HSCT; this may be related to our data, where among the 19 subjects with high VAF of *FLT3*-ITD mutations, 12 were found to have concurrent *DNMT3A* mutations. Several studies have reported *NPM1*/*FLT3*-ITD/*DNMT3A*, the most common triple mutation pattern in *NPM1*-mutated patients, with extremely poor prognosis [9,15,16,17,18], and allo-HSCT has been shown to significantly improve the survival of this subgroup [15].

We further identified that *DDX11* mutations had prognostic significance in our study. DDX11 encodes an iron–sulfur cluster DNA helicase required for development, mutation and over-expression in cancers [31]. *DDX11* has been shown to be associated with the progression of multiple cancers, including melanoma [32], lung adenocarcinoma [33], and hepatocellular carcinoma [34]. In multivariate analyses conducted in a CIBMTR study, it was revealed that *DDX11* mutations affect survival by increasing the risk of both relapse and transplant-related mortality in patients with myelodysplastic syndrome [35]. Encouragingly, this study further validates the recent findings published by us, which indicated that CN-AML patients with *DDX11* mutations have a significantly higher CIR and poorer LFS [36], and *DDX11* dysfunctions were linked to AML via promoting cell proliferation [37]. Other than that, *DDX11* mutations have not been reported in *NPM1*-mutated CN-AML, and the mutation frequency was 8% (N = 12) in our study; nine of these subjects relapsed.

In addition to baseline genetic characterization, the 2022 ELN guidelines also emphasize the importance of response to initial therapy and early assessment of MRD in individual risk assignment [29]. In clinical practice, MRD can serve as an important biomarker for prognosis, prediction, monitoring, and efficacy evaluation [38]. However, MRD-negative patients may still relapse in some cases, while some MRD-positive patients may remain in CR. Additionally, MRD positivity might be more common in genetically adverse AML cases compared to genetically favorable ones, suggesting that MRD is influenced to some extent by genetic factors [39]. Therefore, integrating MRD monitoring results with additional genetic data during the treatment process for risk stratification may further enhance prognostic prediction.

Our study has important limitations. First, it was retrospective study. Second, the subjects received diverse therapies and were not randomly assigned to receive a transplant. Third, our censoring of transplant recipients was imperfect. Fourth, there were few subjects with *DDX11* gene mutations. Given these limitations, our conclusions need validation. Our data, if validated, could assist physicians in predicting the prognostic risk stratification for subjects with CN-AML and *NPM1* mutations, and could potentially be incorporated into clinical decision-making.

## Figures and Tables

**Table 1:** Subject co-variates and post-remission therapy.

	No Transplant (N = 99)	Transplant (N = 51)	*p*-Value
Gender (male/female)	48/51	30/21	0.30
Age (median, range)	53 (16–76)	33 (17–61)	<0.001
WBC (×10^9^/L) (median, range)	29.8 (0.3–224.4)	35.1 (0.9–266.0)	0.69
Hemoglobin (g/L) (mean, SD)	87 (22)	88 (25)	0.86
Platelets (×10^9^/L) (median, range)	68 (6–806)	59 (10–535)	0.61
Bone marrow blasts (%) (median, range)	72 (26–99)	70 (21–96)	0.31
CR after first induction (*n*, %)	78 (79%)	44 (86%)	0.28
MRD-positive (*n*, %)	50 (51%)	23 (45%)	0.61

Abbreviations: CR, complete remission; MRD, measurable residual disease.

**Table 2:** Associations of mutations with subject-related variables.

Variable	*DNMT3A*		*FLT3*-ITD		*IDH2*		*PTPN11*	
	WT (N = 76)	MUT (N = 74)	WT (N = 98)	MUT (N = 52)	WT (N = 106)	MUT (N = 44)	WT (N = 121)	MUT (N = 29)
Gender, male/female	35/41	43/31	48/50	30/22	53/53	25/19	62/59	16/13
Age (median, range)	41 (17–73)	50 (22–76) *	49 (17–76)	47 (16–68)	48 (16–76)	48 (17–65)	47 (16–76)	48 (17–67)
WBC (×10^9^/L) (median, range)	21 (0–228)	37 (2–226) *	29 (0–266)	38 (1–185)	32 (1–266)	25 (0–192)	31 (0–266)	30 (3–224)
Hemoglobin (g/L) (mean, SD)	85 (24)	91(23)	85 (23)	92 (24)	86 (24)	91 (22)	88 (24)	86 (19)
Platelets (×10^9^/L) (median, range)	48 (6–267)	76(11–806) *	69 (6–535)	56(10–806)	59 (6–806)	78 (7–535)	64 (6–806)	73 (9–211)
Bone marrow blasts (%) (median, range)	70 (23–96)	72 (21–99)	65 (21–99)	76 (27–96) *	70 (21–94)	73 (31–99)	71 (24–99)	57 (21–91)

WT, wild type; MUT, mutation; SD, standard deviation. * *p* < 0.05 when compared with wild type.

**Table 3:** Multivariate analyses of CIR and LFS.

Variables	Multivariate Analysis of CIR		Multivariate Analysis of LFS	
	HR (95% CI)	*p*-Value	HR (95% CI)	*p*-Value
MRD test (+/−)	6.00 (3.31–10.85)	<0.001	5.49 (3.01–10.04)	<0.001
*DNMT3A* (Mutated vs. WT)	3.01 (1.57–5.78)	<0.001	2.99 (1.60–5.62)	<0.001
*FLT3*-ITD (Mutated with VAF ≥ 0.5 vs. WT or mutated with VAF < 0.5)	4.40 (1.89–10.24)	<0.001	4.20 (1.87–9.40)	<0.001
*DDX11* (Mutated vs. WT)	4.38 (2.38–8.04)	<0.001	4.22 (1.99–8.95)	<0.001

MRD, measurable residual disease; WT, wild type; VAF, variant allele frequency; CIR, cumulative incidence of relapse; HR, hazard ratio; CI, confidence interval; LFS, leukemia-free survival.

## Data Availability

The original contributions presented in this study are included in the article/Supplementary Materials. Further inquiries can be directed to the corresponding authors.

## Supplementary Materials

The following supporting information can be downloaded at: https://www.mdpi.com/article/10.3390/biomedicines12122921/s1, Figure S1: Cumulative incidence of relapse (A) and leukemia-free survival (B) of post-remission therapies; Figure S2: Variant allele frequencies (VAFs) of different driver gene mutations; Figure S3: Genomic analysis. (A) Pair-wise association between genes mutated in ≥10 subjects. (B) KEGG enrichment analysis; Figure S4: Cumulative incidence of relapse (A) and leukemia- free survival (B) in subjects with *DDX11* mutations versus wild type; Table S1: The 236-gene panel for TRS; Table S2: KEGG pathway enrichment analysis; Table S3: Univariate analyses of CIR and LFS.

## References

[1] Tian X., Xu Y., Yin J., Tian H., Chen S., Wu D., Sun A. (2014). *TET2* gene mutation is unfavorable prognostic factor in cytogenetically normal acute myeloid leukemia patients with *NPM1*+ and *FLT3*-ITD-mutations. Int. J. Hematol..

[2] Wang M., Yang C., Zhang L., Schaar D.G. (2017). Molecular Mutations and Their Cooccurrences in Cytogenetically Normal Acute Myeloid Leukemia. Stem Cells Int..

[3] Papaemmanuil E., Gerstung M., Bullinger L., Gaidzik V.I., Paschka P., Roberts N.D., Potter N.E., Heuser M., Thol F., Bolli N. (2016). Genomic Classification and Prognosis in Acute Myeloid Leukemia. N. Engl. J. Med..

[4] Thiede C., Koch S., Creutzig E., Steudel C., Illmer T., Schaich M., Ehninger G. (2006). Prevalence and prognostic impact of *NPM1* mutations in 1485 adult patients with acute myeloid leukemia (AML). Blood.

[5] Khoury J.D., Solary E., Abla O., Akkari Y., Alaggio R., Apperley J.F., Bejar R., Berti E., Busque L., Chan J.K.C. (2022). The 5th edition of the World Health Organization Classification of Haematolymphoid Tumours: Myeloid and Histiocytic/Dendritic Neoplasms. Leukemia.

[6] Falini B., Brunetti L., Sportoletti P., Martelli M.P. (2020). *NPM1*-mutated acute myeloid leukemia: From bench to bedside. Blood.

[7] Boddu P., Kantarjian H., Borthakur G., Kadia T., Daver N., Pierce S., Andreeff M., Ravandi F., Cortes J., Kornblau S.M. (2017). Co-occurrence of *FLT3*-TKD and *NPM1* mutations defines a highly favorable prognostic AML group. Blood Adv..

[8] Eisfeld A.K., Kohlschmidt J., Mims A., Nicolet D., Walker C.J., Blachly J.S., Carroll A.J., Papaioannou D., Kolitz J.E., Powell B.E. (2020). Additional gene mutations may refine the 2017 European LeukemiaNet classification in adult patients with de novo acute myeloid leukemia aged <60 years. Leukemia.

[9] Bezerra M.F., Lima A.S., Piqué-Borràs M.R., Silveira D.R., Coelho-Silva J.L., Pereira-Martins D.A., Weinhäuser I., Franca-Neto P.L., Quek L., Corby A. (2020). Co-occurrence of *DNMT3A, NPM1, FLT3* mutations identifies a subset of acute myeloid leukemia with adverse prognosis. Blood.

[10] Xu L., Chen H., Chen J., Han M., Huang H., Lai Y., Liu D., Liu Q., Liu T., Jiang M. (2018). The consensus on indications, conditioning regimen, and donor selection of allogeneic hematopoietic cell transplantation for hematological diseases in China-recommendations from the Chinese Society of Hematology. J. Hematol. Oncol..

[11] Wang Y., Chang Y.J., Chen J., Han M., Hu J., Hu J., Huang H., Lai Y., Liu D., Liu Q. (2024). Consensus on the monitoring, treatment, and prevention of leukaemia relapse after allogeneic haematopoietic stem cell transplantation in China: 2024 update. Cancer Lett..

[12] Zhou Y.L., Wu L.X., Peter Gale R., Wang Z.L., Li J.L., Jiang H., Jiang Q., Jiang B., Cao S.B., Lou F. (2020). Mutation topography and risk stratification for de novo acute myeloid leukaemia with normal cytogenetics and no nucleophosmin 1 (*NPM1*) mutation or Fms-like tyrosine kinase 3 internal tandem duplication (*FLT3*-ITD). Br. J. Haematol..

[13] Ruan G.R., Li J.L., Qin Y.Z., Li L.D., Xie M., Chang Y., Zhang Y., Liu Y.R., Jiang B., Chen S.S. (2009). Nucleophosmin mutations in Chinese adults with acute myelogenous leukemia. Ann. Hematol..

[14] Zhu H.H., Zhang X.H., Qin Y.Z., Liu D.H., Jiang H., Chen H., Jiang Q., Xu L.P., Lu J., Han W. (2013). MRD-directed risk stratification treatment may improve outcomes of t(8;21) AML in the first complete remission: Results from the AML05 multicenter trial. Blood.

[15] Yao Y., Zhou Y., Zhuo N., Xie W., Meng H., Lou Y., Mao L., Tong H., Qian J., Yang M. (2024). Co-mutation landscape and its prognostic impact on newly diagnosed adult patients with *NPM1*-mutated de novo acute myeloid leukemia. Blood Cancer J..

[16] Park D.J., Kwon A., Cho B.S., Kim H.J., Hwang K.A., Kim M., Kim Y. (2020). Characteristics of *DNMT3A* mutations in acute myeloid leukemia. Blood Res..

[17] Mason E.F., Hasserjian R.P., Aggarwal N., Seegmiller A.C., Pozdnyakova O. (2019). Blast phenotype and comutations in acute myeloid leukemia with mutated *NPM1* influence disease biology and outcome. Blood Adv..

[18] Gale R.E., Lamb K., Allen C., El-Sharkawi D., Stowe C., Jenkinson S., Tinsley S., Dickson G., Burnett A.K., Hills R.K. (2015). Simpson’s Paradox and the Impact of Different *DNMT3A* Mutations on Outcome in Younger Adults With Acute Myeloid Leukemia. J. Clin. Oncol..

[19] Gaidzik V.I., Schlenk R.F., Paschka P., Stölzle A., Späth D., Kuendgen A., von Lilienfeld-Toal M., Brugger W., Derigs H.G., Kremers S. (2013). Clinical impact of *DNMT3A* mutations in younger adult patients with acute myeloid leukemia: Results of the AML Study Group (AMLSG). Blood.

[20] Brunetti L., Gundry M.C., Goodell M.A. (2017). DNMT3A in Leukemia. Cold Spring Harb. Perspect. Med..

[21] Marcucci G., Metzeler K.H., Schwind S., Becker H., Maharry K., Mrózek K., Radmacher M.D., Kohlschmidt J., Nicolet D., Whitman S.P. (2012). Age-related prognostic impact of different types of *DNMT3A* mutations in adults with primary cytogenetically normal acute myeloid leukemia. J. Clin. Oncol..

[22] Sasaki K., Kanagal-Shamanna R., Montalban-Bravo G., Assi R., Jabbour E., Ravandi F., Kadia T., Pierce S., Takahashi K., Nogueras Gonzalez G. (2020). Impact of the variant allele frequency of *ASXL1, DNMT3A, JAK2, TET2, TP53,* and *NPM1* on the outcomes of patients with newly diagnosed acute myeloid leukemia. Cancer.

[23] Ribeiro A.F., Pratcorona M., Erpelinck-Verschueren C., Rockova V., Sanders M., Abbas S., Figueroa M.E., Zeilemaker A., Melnick A., Löwenberg B. (2012). Mutant *DNMT3A*: A marker of poor prognosis in acute myeloid leukemia. Blood.

[24] Tie R., Zhang T., Fu H., Wang L., Wang Y., He Y., Wang B., Zhu N., Fu S., Lai X. (2014). Association between *DNMT3A* mutations and prognosis of adults with de novo acute myeloid leukemia: A systematic review and meta-analysis. PLoS ONE.

[25] Patel J.P., Gönen M., Figueroa M.E., Fernandez H., Sun Z., Racevskis J., Van Vlierberghe P., Dolgalev I., Thomas S., Aminova O. (2012). Prognostic relevance of integrated genetic profiling in acute myeloid leukemia. N. Engl. J. Med..

[26] Kottaridis P.D., Gale R.E., Frew M.E., Harrison G., Langabeer S.E., Belton A.A., Walker H., Wheatley K., Bowen D.T., Burnett A.K. (2001). The presence of a FLT3 internal tandem duplication in patients with acute myeloid leukemia (AML) adds important prognostic information to cytogenetic risk group and response to the first cycle of chemotherapy: Analysis of 854 patients from the United Kingdom Medical Research Council AML 10 and 12 trials. Blood.

[27] Thiede C., Steudel C., Mohr B., Schaich M., Schäkel U., Platzbecker U., Wermke M., Bornhäuser M., Ritter M., Neubauer A. (2002). Analysis of *FLT3*-activating mutations in 979 patients with acute myelogenous leukemia: Association with FAB subtypes and identification of subgroups with poor prognosis. Blood.

[28] Fröhling S., Schlenk R.F., Breitruck J., Benner A., Kreitmeier S., Tobis K., Döhner H., Döhner K. (2002). Prognostic significance of activating *FLT3* mutations in younger adults (16 to 60 years) with acute myeloid leukemia and normal cytogenetics: A study of the AML Study Group Ulm. Blood.

[29] Döhner H., Wei A.H., Appelbaum F.R., Craddock C., DiNardo C.D., Dombret H., Ebert B.L., Fenaux P., Godley L.A., Hasserjian R.P. (2022). Diagnosis and management of AML in adults: 2022 recommendations from an international expert panel on behalf of the ELN. Blood.

[30] Stone R.M. (2018). What FLT3 inhibitor holds the greatest promise?. Best. Pract. Res. Clin. Haematol..

[31] Jegadesan N.K., Branzei D. (2021). DDX11 loss causes replication stress and pharmacologically exploitable DNA repair defects. Proc. Natl. Acad. Sci. USA.

[32] Bhattacharya C., Wang X., Becker D. (2012). The DEAD/DEAH box helicase, DDX11, is essential for the survival of advanced melanomas. Mol. Cancer.

[33] Li J., Liu L., Liu X., Xu P., Hu Q., Yu Y. (2019). The Role of Upregulated DDX11 as A Potential Prognostic and Diagnostic Biomarker in Lung Adenocarcinoma. J. Cancer.

[34] Yu Y., Zhao D., Li K., Cai Y., Xu P., Li R., Li J., Chen X., Chen P., Cui G. (2020). E2F1 mediated *DDX11* transcriptional activation promotes hepatocellular carcinoma progression through PI3K/AKT/mTOR pathway. Cell Death Dis..

[35] Zhang T., Auer P., Dong J., Cutler C., Dezern A.E., Gadalla S.M., Deeg H.J., Nazha A., Carlson K.S., Spellman S. (2023). Whole-genome sequencing identifies novel predictors for hematopoietic cell transplant outcomes for patients with myelodysplastic syndrome: A CIBMTR study. J. Hematol. Oncol..

[36] Zhou Y.L., Zhao M.Y., Gale R.P., Jiang H., Jiang Q., Liu L.X., Qin J.Y., Cao S.B., Lou F., Xu L.P. (2024). Mutations in DEAD/H-box helicase 11 correlate with increased relapse risk in adults with acute myeloid leukaemia with normal cytogenetics. Leukemia.

[37] Zhou Y.-L., Wu L.-X., Gale R.P., Wang Z.-L., Li J.-L., Jiang H., Jiang Q., Jiang B., Cao S.-B., Sun Y. (2019). Dead/H-Box Helicase 11 (*DDX11*) Mutations Correlate with Increased Relapse Risk in Persons with Acute Myeloid Leukaemia and Promote Proliferation and Survival of Human AML Cells in Vitro and in Immune Deficient Mice. Blood.

[38] Heuser M., Freeman S.D., Ossenkoppele G.J., Buccisano F., Hourigan C.S., Ngai L.L., Tettero J.M., Bachas C., Baer C., Béné M.C. (2021). 2021 Update on MRD in acute myeloid leukemia: A consensus document from the European LeukemiaNet MRD Working Party. Blood.

[39] Vidriales M.B., Pérez-López E., Pegenaute C., Castellanos M., Pérez J.J., Chandía M., Díaz-Mediavilla J., Rayón C., de Las Heras N., Fernández-Abellán P. (2016). Minimal residual disease evaluation by flow cytometry is a complementary tool to cytogenetics for treatment decisions in acute myeloid leukaemia. Leuk. Res..

