# Supplementary material for: Acute Myeloid Leukemia with Normal Cytogenetics and NPM1-Mutation: Impact of Mutation Topography on Outcomes
Source: Biomedicines. 2024 Dec 23;12(12):2921. doi: 10.3390/biomedicines12122921 (PMC11673242; doi:10.3390/biomedicines12122921)
Supplement: Supplementary file 1 [file biomedicines-12-02921-s001.zip › Supplement Figures.pdf]

Supplement Figure

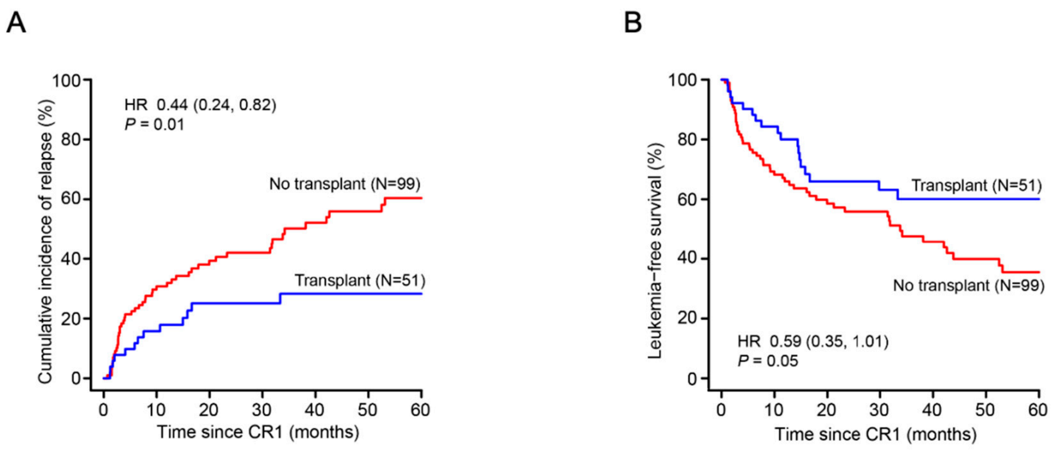

Supplement Figure S1. Cumulative incidence of relapse (A) and leukemia-free survival (B) of post-remission therapies.

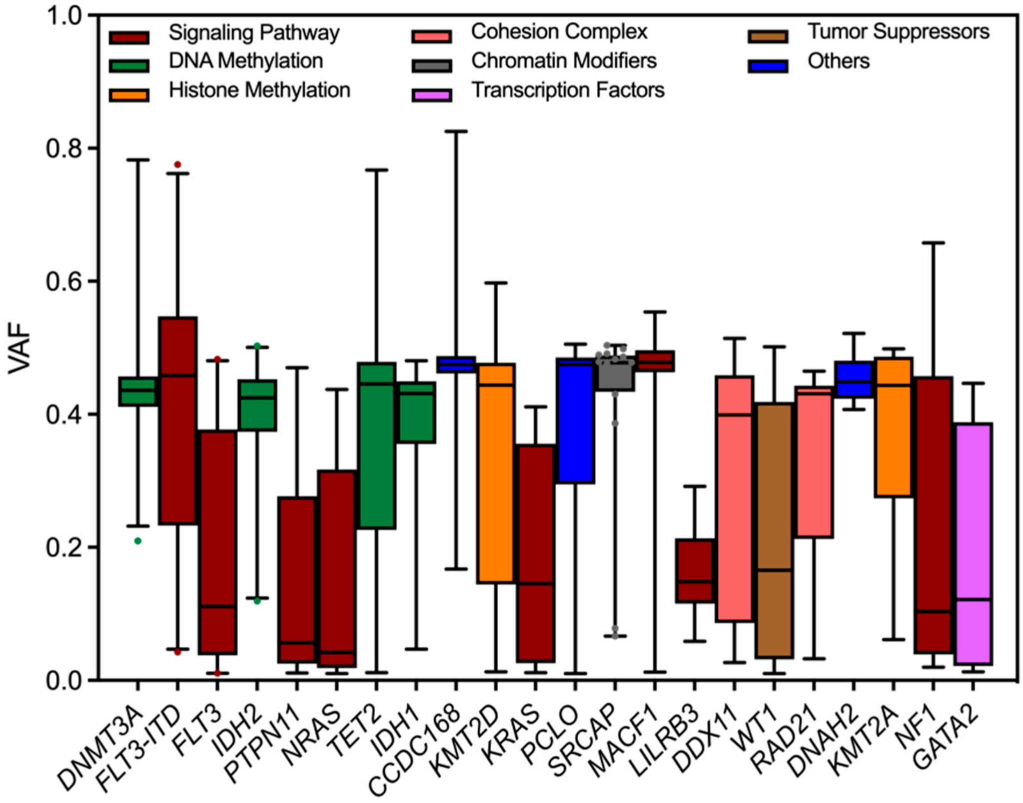

Supplement Figure S2. Variant allele frequencies (VAFs) of different driver gene mutations. Genes mutated in ≥10 subjects are shown. The boxplot shows the median, minimum and maximum VAF observed across the entire

cohort. Boxes are coloured by function of each driver mutation.

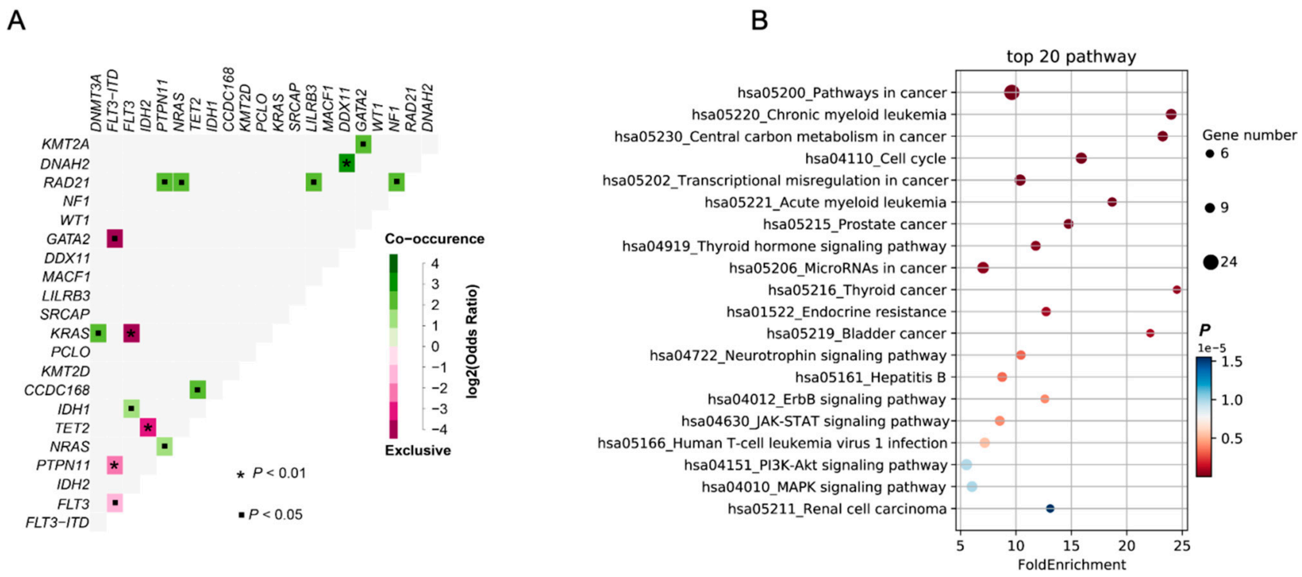

Supplement Figure S3. Genomic analysis. (A) Pair-wise association between genes mutated in ≥ 10 subjects. (B) KEGG enrichment analysis. The top 20 pathways are displayed.

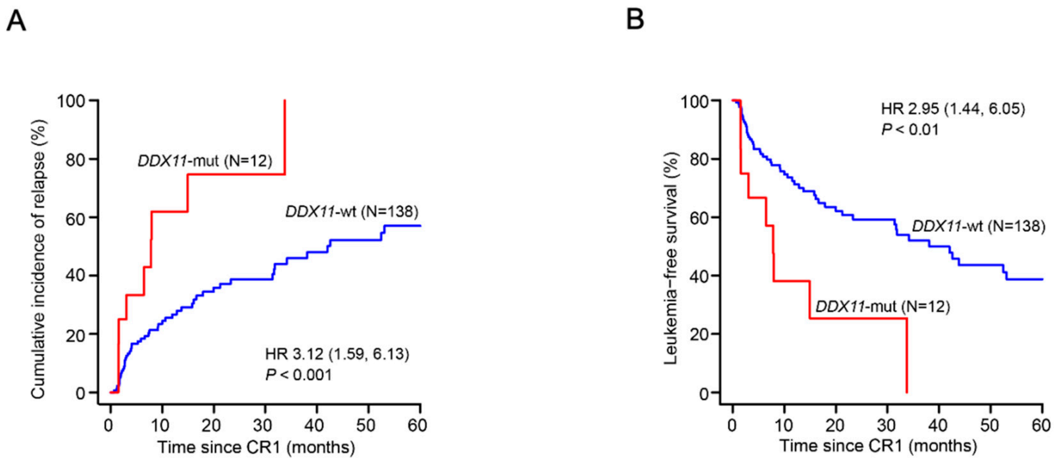

Supplement Figure S4. Cumulative incidence of relapse (A) and leukemia-free survival (B) in subjects with *DDX11* mutations *versus* wild-type.
