# Supplementary material for: Acute Myeloid Leukemia with Normal Cytogenetics and NPM1-Mutation: Impact of Mutation Topography on Outcomes
Source: Biomedicines. 2024 Dec 23;12(12):2921. doi: 10.3390/biomedicines12122921 (PMC11673242; doi:10.3390/biomedicines12122921)
Supplement: Supplementary file 1 [file biomedicines-12-02921-s001.zip › Supplement Tables 1&3.pdf]

Supplement Table S1 The 236-gene panel for TRS

ABCB1	*ABCC3*	*ABCG2*	*ABL1*	*ADSL*	*AKT2*	*AKT3*
ALK	*AMER1*	*ANKRD26*	*ARID1B*	*ARID2*	*ASXL1*	*ASXL2*
ATM	*ATRX*	*B2M*	*BCL11B*	*BCL2*	*BCL6*	*BCOR*
BCORL1	*BIRC3*	*BLM*	*BMP7*	*BRAF*	*BTK*	*CACNA1E*
CACNA1G	*CALR*	*CARD11*	*CBL*	*CCDC168*	*CCND1*	*CCND3*
CD28	*CD58*	*CD79A*	*CD79B*	*CDA*	*CDKN1B*	*CDKN2A*
CEBPA	*CECR2*	*CEP72*	*CHD2*	*CHD8*	*CPA2*	*CREBBP*
CRLF2	*CSF3R*	*CSMD1*	*CTCF*	*CTLA4*	*CTNNB1*	*CUX1*
CXCR4	*CYBA*	*CYP2B6*	*CYP2C19*	*CYP2C8*	*CYP3A4*	*CYP3A5*
DARS	*DCTD*	*DDX41*	*DHX15*	*DHX30*	*DIS3*	*DKC1*
DNAH2	*DNM2*	*DNMT3A*	*DOK5*	*DDX11*	*DYNC2H1*	*EGR2*
ELANE	*EP300*	*EPOR*	*ERCC1*	*ERG*	*ETNK1*	*ETV6*
EVI1	*EZH2*	*FAM46C*	*FAT1*	*FBXW7*	*FCGR3A*	*FGFR1*
FLT3	*FOXO1*	*GATA1*	*GATA2*	*GATA3*	*GFI1*	*GNA13*
GNAS	*GSTM1*	*GSTP1*	*HAX1*	*HIST1H1E*	*HLA-DRB1*	*ID3*
IDH1	*IDH2*	*IKZF1*	*LILRB3*	*IL7R*	*IMPDH2*	*IRF4*
ITPA	*JAK1*	*JAK2*	*JAK3*	*KDM5C*	*KDM6A*	*KDM6B*
KIT	*KMT2A*	*KMT2C*	*KMT2D*	*KRAS*	*LINC00251*	*MACF1*
MAP2K1	*MAP3K7*	*MAPK1*	*MED12*	*MEF2B*	*MLH1*	*MPL*
MSH6	*MTHFR*	*MTRR*	*MYC*	*MYD88*	*NF1*	*NF2*
NFATC2	*NFKBIA*	*NFKBIE*	*NOTCH1*	*NOTCH2*	*NPM1*	*NR3C1*
NRAS	*NSD2*	*NT5C2*	*NTRK1*	*NTRK3*	*NUDT15*	*PAX5*
PCLO	*PDGFRA*	*PDGFRB*	*PHF6*	*PIGA*	*PIK3CA*	*PIK3R1*
PLCG2	*PNPLA3*	*POT1*	*PPM1D*	*PRKDC*	*PROX1-AS1*	*PRPF8*
PRPS1	*PTEN*	*PTPN11*	*RAD21*	*RB1*	*RHOA*	*RIT1*
ROBO1	*ROBO2*	*ROBO3*	*RPL10*	*RPS15*	*RRM1*	*RRM2*
RRM2B	*RUNX1*	*SAMHD1*	*SBDS*	*SERPINE1*	*SETBP1*	*SETD2*
SF1	*SF3B1*	*SH2B3*	*SLC22A1*	*SLC29A1*	*SLCO1A2*	*SLCO1B1*
SMAD4	*SMC1A*	*SMC3*	*SOCS1*	*SOD2*	*SOS1*	*SOX11*
SPI1	*SRCAP*	*SRP72*	*SRSF2*	*STAG1*	*STAG2*	*STAT3*
STAT5A	*STAT5B*	*STAT6*	*STIM1*	*SUZ12*	*TCF3*	*TERC*
TERT	*TET2*	*TNF*	*TNFAIP3*	*TNFRSF14*	*TP53*	*TPMT*
TRAF3	*TRIM24*	*U2AF1*	*UGT1A1*	*UGT1A8*	*USH2A*	*USP7*
WT1	*XPO1*	*XRCC5*	*ZMYM3*	*ZRSR2*		

Supplement Table S3 Uni-variate analyses of CIR and LFS

Variables	uni-variate analysis of CIR	uni-variate analysis of LFS
	P value	*P* value
sex	0.53	0.39
age	0.68	0.74
WBC	0.05	0.08
MRDind	0.88	0.99
MRDcon1	0.01	0.03
MRDcon2	0.00	0.00
DNMT3A	0.00	0.00
FLT3-ITD_VAF	0.00	0.00
IDH2	0.85	0.89
PTPN11	0.50	0.34
NRAS	0.20	0.14
TET2	0.39	0.54
IDH1	1.00	0.83
CCDC168	0.92	0.77
KMT2D	0.88	0.99
KRAS	0.77	0.89
PCLO	0.45	0.56
LILRB3	0.36	0.14
MACF1	0.92	0.80
SRCAP	0.22	0.20
DDX11	0.00	0.00
DNAH2	0.76	0.70
GATA2	0.07	0.08
KMT2A	0.58	0.65
NF1	0.70	0.80
RAD21	0.52	0.60

WT1	0.50	0.44

WBC, white blood cell; MRD (ind / con1 / con2), measurable residual disease (after induction therapy / after the 1st / 2nd consolidation cycle); VAF, variant allele frequency; CIR, cumulative incidence of relapse; LFS, leukemia-free survival.
